# Boston Medical and Surgical Journal

**Published:** 1849-04

**Authors:** 


### BOSTON MEDICAL AND SURGICAL JOURNAL.

The first number of the fortieth volume of this spirited journal lies before us on our table. There is something encourging in the fact, that during these twenty years it has never changed editors, and never appeared to languish; a fact which speaks loudly in favor of the industry and tact of the editor, and of the punctuality of its subscribers. We trust that many years of cloudless prosperity are yet in reserve for our valued, and shall we not say, venerable, contemporary?

